# Simple Nanoparticles from the Assembly of Cationic Polymer and Antigen as Immunoadjuvants

**DOI:** 10.3390/vaccines8010105

**Published:** 2020-02-28

**Authors:** Yunys Pérez-Betancourt, Bianca de Carvalho Lins Fernandes Távora, Mônica Colombini, Eliana L. Faquim-Mauro, Ana Maria Carmona-Ribeiro

**Affiliations:** 1 Biocolloids Laboratory, Departamento de Bioquímica, Instituto de Química, Universidade de São Paulo, São Paulo 05508-000, Brazil; y.betancourt@usp.br; 2 Immunopathology Laboratory, Butantan Institute, São Paulo 05503-900, Brazil; bianca.tavora@butantan.gov.br (B.d.C.L.F.T.); eliana.faquim@butantan.gov.br (E.L.F.-M.); monica.colombini@butantan.gov.br (M.C.)

**Keywords:** cationic polymer, poly (diallyldimethylammonium chloride), model antigen, ovalbumin, antigen/cationic polymer nanoparticles, poly-cation cytotoxicity, dynamic light scattering, scanning electron microscopy, ELISA assays, delayed-type hypersensitivity assay

## Abstract

Since antigens are negatively charged, they combine well with positively charged adjuvants. Here, ovalbumin (OVA) (0.1 mg·mL^−1^) and poly (diallyldimethylammonium chloride) (PDDA) (0.01 mg·mL^−1^) yielded PDDA/OVA assemblies characterized by dynamic light scattering (DLS) and scanning electron microscopy (SEM) as spherical nanoparticles (NPs) of 170 ± 4 nm hydrodynamic diameter, 30 ± 2 mV of zeta-potential and 0.11 ± 0.01 of polydispersity. Mice immunization with the NPs elicited high OVA-specific IgG1 and low OVA-specific IgG2a production, indicating a Th-2 response. Delayed-type hypersensitivity reaction (DTH) was low and comparable to the one elicited by Al(OH)_3_/OVA, suggesting again a Th-2 response. PDDA advantages as an adjuvant were simplicity (a single-component adjuvant), low concentration needed (0.01 mg·mL^−1^ PDDA) combined with antigen yielding neglectable cytotoxicity, and high stability of PDDA/OVA dispersions. The NPs elicited much higher OVA-specific antibodies production than Al(OH)_3_/OVA. In vivo, the nano-metric size possibly assured antigen presentation by antigen-presenting cells (APC) at the lymph nodes, in contrast to the location of Al(OH)_3_/OVA microparticles at the site of injection for longer periods with stimulation of local dendritic cells. In the future, it will be interesting to evaluate combinations of the antigen with NPs carrying both PDDA and elicitors of the Th-1 response.

## 1. Introduction

Adjuvants are essential components of modern vaccines; they enhance the magnitude and guide the type of adaptive immune response to produce the most effective form of immunity for each specific pathogen [1,2]. Adjuvants act, mostly, as antigen carriers or/and as pattern recognition receptors (PRR) agonists. The demand for adjuvants in vaccine formulations emerged from the application of purified antigens, which are poor immunogens due to lack of the danger signals of pathogens’ entire cells, crucial for activating the innate immune system [1,3,4]. Currently, there are few adjuvants licensed for human use; aluminum-based adjuvants like Al(OH)_3_ are the only ones approved worldwide performing the task of presenting negatively charged antigens due to their positive charge at the pH of water [5,6,7]. Other cationic adjuvants based on nanoparticles [8,9,10,11], liposomes [12,13,14], cationic bilayer fragments [8,15,16] or supported cationic bilayers on polymeric nanoparticles (NPs) [9], silica [17], or cationic polymers on superparamagnetic iron oxide NPs [18] have also been proposed as effective micro or nanomaterials able to effectively interact with antigens and antigen-presenting cells (APC). 

Major mechanisms for taking up the cationic assemblies depend on size [19,20]. Nanoparticles with 20–200 nm mean diameter are like viruses and usually taken up by endocytosis via clathrin-coated vesicles, caveolae, or their independent receptors, and preferentially ingested by dendritic cells (DC) [21]. Microparticles with 500–5000 nm diameter are like bacteria, captured by phagocytosis and primarily ingested by macrophages. All particles used in vaccine formulations are consequently internalized efficiently by APC by one or a combination of the quoted mechanisms [22,23]. Particles with diameters smaller than 500 nm, in particular the nano-sized ones with 40–100 nm diameter, are more efficient to promote CD8 and CD4 type 1 T-helper cells responses than those with diameters above 500 nm; large particles, more similar to Al (OH)_3_, usually induce good T-helper cells type 2 improvement in antibody responses [22]. In summary, cationic micro and nanoparticles are effectively taken up both by macrophages and dendritic cells since electrostatic attraction promotes particle binding and subsequent internalization [11,14,20,24,25]. Cationic particles and liposomes containing dioctadecyldimethylammonium bromide (DODAB) cationic lipid [13,26] carrying *Mycobacterium tuberculosis* [13,27], *Chlamydia trachomatis* [11], or *Neisseria meningitides* antigens enhanced the cellular and humoral immune response against them [16,28]. 

Another important class of cationic adjuvants is represented by the cationic polymers despite their overt cytotoxicity [29,30,31,32,33]. They easily combine with oppositely charged proteins [34]. Particles of poly (lactic-co-glycolic acid) (PLGA) have been combined with cationic polymers like polyethyleneimine (PEI) or ε-poly-L-lysine (PLL) for improving antigen adsorption, colloidal stability, and the immune response [35]. Nanocomplexes of PEI and antigens (influenza hemagglutinin or herpes simplex virus type-2 glycoprotein D) delivered by the mucosal route activated APC in vivo, promoting dendritic cell trafficking to draining lymph nodes besides and eliciting a potent immune response against the viral subunit glycoproteins; a single intranasal administration elicited robust antibody-mediated protection [36]. Systemic administration of the same antigens with PEI induced both Th1/Th2 immune responses and higher titers of both antigen-binding and -neutralizing antibodies than alum [37]. 

The cationic antimicrobial polymer poly (diallyldimethylammonium chloride) (PDDA) is a poly-cation [38,39,40] able to combine with bovine serum albumin (BSA), yielding NPs with diameters around 50 nm [41,42]. In combination with HIV-1 DNA, nanorods of gold yielded particles of gold/ PDDA/DNA, which elicited a Th-2 response that was higher than the one obtained using PEI or cetyltrimethylammonium bromide (CTAB). Stimulated cellular and humoral immunity, as well as T cell proliferation, were obtained in comparison with naked HIV-1 Env plasmid DNA treatment in vivo [43]. 

In this work, NPs of PDDA/ovalbumin are prepared, characterized by their physical properties, and evaluated as stimulators of the OVA-specific immune response. The results obtained by means of dynamic light scattering (DLS) show that cationic PDDA/OVA NPs at 0.01 and 0.1 mg·mL^−1^ PDDA and OVA, respectively, have 170 ± 4 nm of hydrodynamic diameter, 30 ± 2 mV of zeta-potential, and 0.11 ± 0.01 of polydispersity. These NPs used for mice immunization elicited potent Th-2 OVA-specific immune response. There were high OVA-specific IgG1 and low OVA-specific IgG2a production. Delayed-type hypersensitivity reaction (DTH) was low and comparable to the one elicited by Al(OH)_3_/OVA, suggesting again a Th-2 response. PDDA advantages as an adjuvant are simplicity (a single-component adjuvant), low concentration needed (0.01 mg·mL^−1^ PDDA) combined with antigen yielding neglectable cytotoxicity, and high stability of PDDA/OVA dispersions. The NPs elicited much higher OVA-specific antibody production than Al(OH)_3_/OVA. 

## 2. Materials and Methods 

### 2.1. Reagents and Stock Solutions

Poly (diallyldimethylammonium chloride) (PDDA), grade V ovalbumin (OVA), peroxidase-labeled streptavidin, and o-phenylenediamine (OPD) were purchased from Sigma Chemical Co. (St. Louis, MO, USA). OVA stock solutions (20 mg·mL^−1^) were prepared in Milli-Q water (pH = 6.3) and purified to reduce endotoxin levels by means of a chromatographic procedure using polymyxin B columns (Pierce Biotechnology, Rockford, IL, USA). The concentration of purified OVA in the stock solution was determined by the bicinchoninic acid assay (BCA) [44] and adjusted to 10 mg·mL^−1^. Aluminum hydroxide (Al(OH)_3_) was purchased from Sanofi-Synthelabo, RJ, Brazil. PDDA molecular weight was <100,000 g and it was available as a water solution at 35% PDDA. 

### 2.2. Preparation of the PDDA/OVA Nanoparticles (NPs)

PDDA/OVA NPs were obtained from aliquots of stock PDDA (10 mg·mL^−1^) and OVA (10 mg·mL^−1^) solutions vortexed for 30 s in Milli-Q water at room temperature. The turbid dispersions of NPs with good colloidal stability were used 1 h after preparation. 

### 2.3. Determination of Physical Properties and Colloidal Stability for NPs Dispersions from Dynamic Light Scattering (DLS), Turbidimetry, and Scanning Electron Microscopy (SEM)

All dispersions were obtained in Milli-Q water. Size distribution, zeta-average diameter (Dz), zeta-potential (ζ), polydispersity (P), conductance (G), and colloidal stability for the dispersions were determined by DLS using a Zeta-Plus Zeta-Potential Analyzer (Brookhaven Instruments Corporation, Holtsville, NY) equipped with a 677 nm laser. The diameter from DLS is the mean hydrodynamic (zeta-average) diameter Dz unless otherwise stated. Zeta-potential (ζ) was determined from the electrophoretic mobility μ and Smoluchowski equation, ζ = μη/ε, where η and ε are the viscosity and the dielectric constant of the medium, respectively. The physical properties of the dispersions (Dz, ζ, and polydispersity P) from the DLS technique were determined by applying well-defined mathematical equations [45]. The colloidal stability of the dispersions (at 0.01 and 0.1 mg·mL^−1^ of PDDA and OVA concentrations, respectively) was followed for 48 h by determining the effect of time on Dz, P, ζ, and G.

UV–Vis spectra of the PDDA/OVA dispersions were acquired at 25 °C using a Shimadzu UV-1800 spectrophotometer (SSI; Kyoto, Japan) from 200 to 800 nm in appropriate cuvettes against a Milli-Q blank of pure water. 

For SEM experiments, the PDDA/OVA NPs dispersion in Milli-Q water (at 0.01 and 0.1 mg·mL^−1^ of PDDA and OVA concentrations, respectively) was placed on a round glass coverslip and dried overnight at room temperature before coating with a gold layer using a Leica EM SCD 050 sputtering apparatus for observation under a FEI Quanta 250 scanning electron microscope.

### 2.4. Cell Culture, Preparation of PDDA, OVA, or PDDA/OVA Solutions for Interaction with Cells, and Determination of Cell Viability in the Interaction Mixtures

L929 fibroblasts and J774A.1 macrophages cell lines were obtained from the American Type Culture Collection (ATCC; www.atcc.org) and cultured according to standard protocols for mammalian cells culture under sterile conditions, in an atmosphere of 90% humidity, 5% CO_2_, at 37 °C in RPMI-1640 medium supplemented with 10% fetal bovine serum (FBS), 1 unit/mL of penicillin-streptomycin, and 2 mM L-glutamine. For interaction with cells, PDDA, OVA, and PDDA/OVA solutions were also prepared in RPMI-1640 medium supplemented with 10% fetal bovine serum (FBS), 1 unit/mL of penicillin-streptomycin, and 2 mM L-glutamine. The interaction between cells and solutions to be tested was performed as follows.

L929 fibroblasts and J774A.1 macrophages were plated into 96-well microtiter plates at a density of 10,000 cells/well and incubated for 12 h before replacing the culture medium by 100 µL of PDDA, OVA, or PDDA/OVA solutions. Thereafter, the mixtures were again incubated in a humidified CO_2_ incubator for 3 and 24 h before determining the in vitro cytotoxicity of PDDA, OVA, and PDDA/OVA by the 3-(4,5-dimethylthiazol-2-yl)-2,5-diphenyl tetrazolium bromide (MTT) assay [46]. A MTT stock solution at 5 mg·mL^−1^ in PBS was filtered for sterilization and removal of insoluble MTT residues. Ten microliters of this stock MTT solution was added per well, each well containing 100 µL of the plated cells interacting with OVA, PDDA, PDDA/OVA. After incubation (37 °C/ 2 h), the supernatants were withdrawn and cells adhered to the wells were mixed meticulously with 100 µL of an isopropanol solution acidified to 0.04 N HCl in order to dissolve formazan crystals. The absorbance of each well was recorded at 570 nm on a Multiskan Ex Microelisa reader. As a control, cells mixed with the culture medium only yielded 100% of cell viability. Cell viability in the presence of OVA, PDDA, or OVA/PDDA solutions was expressed as % of the control.

### 2.5. Determination of Cells Morphology in the Presence of PDDA by Scanning Electron Microscopy (SEM)

L929 fibroblasts (ATCC CCL-1) were grown up to 80–100% confluency before taking aliquots of this culture to counting in a Neubauer chamber to add about 100,000 cells/well in wells that had received spherical coverslips (one coverslip per well) in plates with 24 wells/plate so that cells were allowed to adhere on the coverslips for 12 h. The culture medium was replaced by PDDA solutions at concentrations of 0.01, 0.1, and 1 mg·mL^−1^, and the samples were incubated in a humidified CO_2_ incubator for 3 h. Cells were then fixed in the Karnovsky buffer (5% glutaraldehyde, paraformaldehyde 4%, sodium cacodylate 0.1 M, and pH 7.2) for 3h before progressive dehydration was performed with added ethanol solutions (concentrations ranging from 7.5% to 100%; 15 min interaction with cells per concentration). Thereafter, the cells on the coverslips were further dried in a Leica CPD 030 critical-point dryer device and covered with gold in a Leica EM SCD 050 sputtering device for observation in an FEI Quanta 250 scanning electron microscope.

### 2.6. Immunization Scheme 

Groups of five BALB/c mice were immunized subcutaneously at two separate sites on the base of the tail with a total injection volume of 0.2 mL/animal using solutions or dispersions at the final concentrations of adjuvants and/or OVA antigen shown in Table 1. A booster using the same dose employed for priming was carried out at 21 days post-immunization.

Ethical procedures for experimentation with animals were followed and were in accordance with guidelines approved by the Instituto Butantan’s Committee of Ethics on Animal Research (protocol number 7912280219). 

### 2.7. Determination of OVA-Specific IgG1 and IgG2a Antibodies

For evaluation of the humoral response, five mice per group were bled through the ophthalmic plexus 14, 21, and 28 days after immunization and the serum individually analyzed by indirect enzyme-linked immunosorbent assay (ELISA). Briefly, 96-well polystyrene high-binding microtitre plates (Costar Corning Inc., Mass., USA) were coated overnight at 4 °C with 100 μL of OVA solution (10 μg/mL in 0.01 M phosphate buffer saline (PBS), pH 7.2). The wells were blocked with 3% gelatin in PBS for 2 h and then incubated (1 h/37 °C) with serum samples serially diluted. In each well, 100 μL of goat anti-mouse biotin-conjugated IgG1 (1:1000) or IgG2a (1:500) (Southern Biotechnology Associates, AL, USA) were added to the corresponding plates and incubated for 1 h at 37 °C. Then, peroxidase-labeled streptavidin (100 μL of a 1:3000 dilution) was added and incubated for 1 h at 37 °C. Plates were washed three times after each incubation step with PBS containing 0.05% Tween 20 (PBST). Finally, 100 μL OPD substrate solution (1 mg·mL^−1^) and H_2_O_2_ (1 μL/mL) diluted in 0.1 M citrate-phosphate (pH 5.0) were added to each well and incubated for 15 min at room temperature. The reaction was stopped by adding 50 μL of 2 M H_2_SO_4_ to each well. Absorbance was determined at 492 nm for individual wells using an ELISA plate reader (Multiskan Ex, Thermo Electron Corporation). The results were expressed for diluted serum samples as the mean absorbance ± standard deviation. The primary response of IgG1 was presented at dilution of 1/256 and the secondary response at 1/16348. The primary response of IgG2a was presented at dilutions 1/8 and the secondary response at 1/128, the dilutions chosen correspond to the linear part of the titration curve.

### 2.8. Determination of Delayed-Type Hypersensitivity Reaction (DTH) 

For evaluation of the delayed-type hypersensitivity reaction, five mice/group on the fifth day post-immunization were injected at the left-hind footpad with a previously heated (1 h/80 °C) and denaturated OVA solution (30 μL, 2 μg/μL in saline). As a control, the same volume of saline was injected at the right-hind footpad of each animal. Differential footpad swelling was determined 24 h after injection with a Mitutoyo digital micrometer and considered as the difference between swelling of left and right paws for the same animal. The results were represented as arithmetic mean footpad swelling ± standard deviation. 

### 2.9. Statistical Analysis

To compare results for different groups, two-way analysis of variance (ANOVA) followed by Tukey’s multiple comparisons test was used. P-values below 0.05 were considered significant. Statistical analysis was performed using the Origin 2018 program (Origin Lab Corporation, Northampton, USA). Statistical analyses for IgG1 or IgG2a were performed separately.

## 3. Results and Discussion

### 3.1. Characterization of PDDA/OVA Dispersions Regarding Formation of Nanoparticles, Their Size, Surface Potential, Polydispersity, Morphology, and Colloidal Stability.

**Figure 1:**
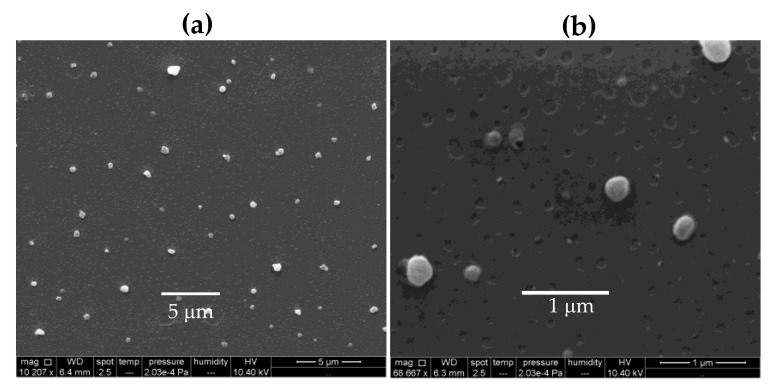
Scanning electron microscopy (SEM) of poly (diallyldimethylammonium chloride) (PDDA)/ ovalbumin (OVA) nanoparticles (NPs) at [PDDA] = 0.01 mg·mL^−1^ and [OVA] = 0.1 mg·mL^−1^ under low (**a**) and high magnification (**b**). The mean diameter (D) of dried NPs from ImageJ software was D = 234 ± 42 nm.

**Figure 2:**
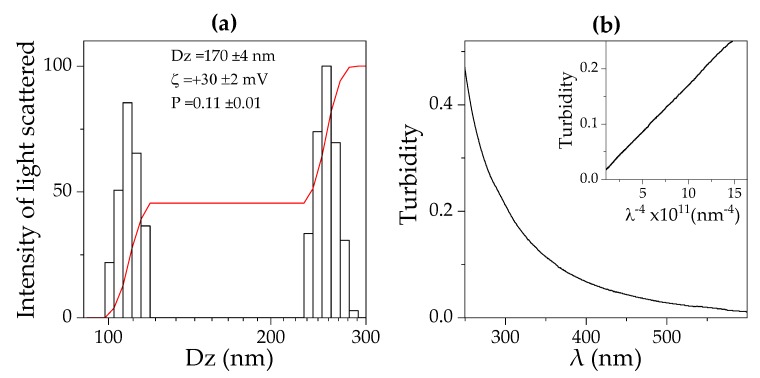
Intensity of light scattered by PDDA/OVA NPs in water obtained at 0.01 PDDA and 0.1 mg·mL^−1^ OVA: (**a**) as a function of the hydrodynamic diameter (Dz) determined by dynamic light scattering (DLS); (**b**) as a function of wavelength of the incident light (λ) determined from turbidimetry. Mean Dz, zeta potential (ζ), and polydispersity (P) for this dispersion are also given. The relationship between turbidity and λ^−4^ was linear with a regression coefficient of 0.90 (insert).

In order to ascertain the formation of nanoparticles from PDDA/OVA dispersions, the first property of the mixtures inferred by visual observation was the occurrence of a turbid appearance. The interaction between OVA and PDDA was driven by their opposite charges. OVA isoelectric point is around 4.5 [47]; its charge at the pH of water (6.3) is negative. PDDA, as a cationic antimicrobial polymer [40], is expected to interact electrostatically with OVA. In fact, PDDA and BSA and other negatively charged proteins interacted in dispersion to yield nanoparticles [41,42,48]. Figure 1 shows micrographs of PDDA/OVA dispersions obtained by SEM. These revealed the occurrence of NPs with a mean diameter (D) of dried NPs of D = 234 ± 42 nm as derived from ImageJ software. Typically, some aggregation due to the drying process (required by the SEM technique) might have slightly increased the mean size of the NPs. Indeed, using a method as DLS, the mean hydrodynamic diameter of the same NPs dispersion was Dz = 170 ± 4 nm (Figure 2a). The NPs at 0.1 mg·mL^−1^ OVA and 0.01 mg·mL^−1^ PDDA displayed a positive net charge (ζ = 30 ± 2), and the dispersion of NPs exhibited a relatively low polydispersity of P = 0.11 ± 0.01.

Curiously, the concentration of the OVA stock solution used to prepare the PDDA/OVA dispersions was important to define the PDDA/OVA NPs size (not shown). The lower the OVA stock solution concentration, the lower the size of PDDA/OVA NPs, possibly due to variable degrees of intermolecular OVA/OVA aggregation. At high concentrations, OVA/OVA aggregation in the stock solution would be higher than at low concentrations. In the literature, OVA/OVA aggregation with increasing OVA concentration has already been observed by other authors [49]. 

The nano-size of all PDDA/OVA NPs in water dispersion was reconfirmed by determining the dependence of turbidity on the wavelength of the incident light (λ). Rayleigh scattering by turbid dispersions of NPs means a linear dependence of turbidity with 1/λ^4^ as the one shown in Figure 2b. Usually, Rayleigh scattering occurs for spherical particles in water dispersion with diameters very small compared with λ [50,51], showing that the PDDA/OVA NPs are indeed spherical and nano-sized.

We defined 0.1 and 0.01 mg·mL^−1^ as the OVA and PDDA concentrations, respectively, to be used throughout this study based on the experiments described next.

**Figure 3:**
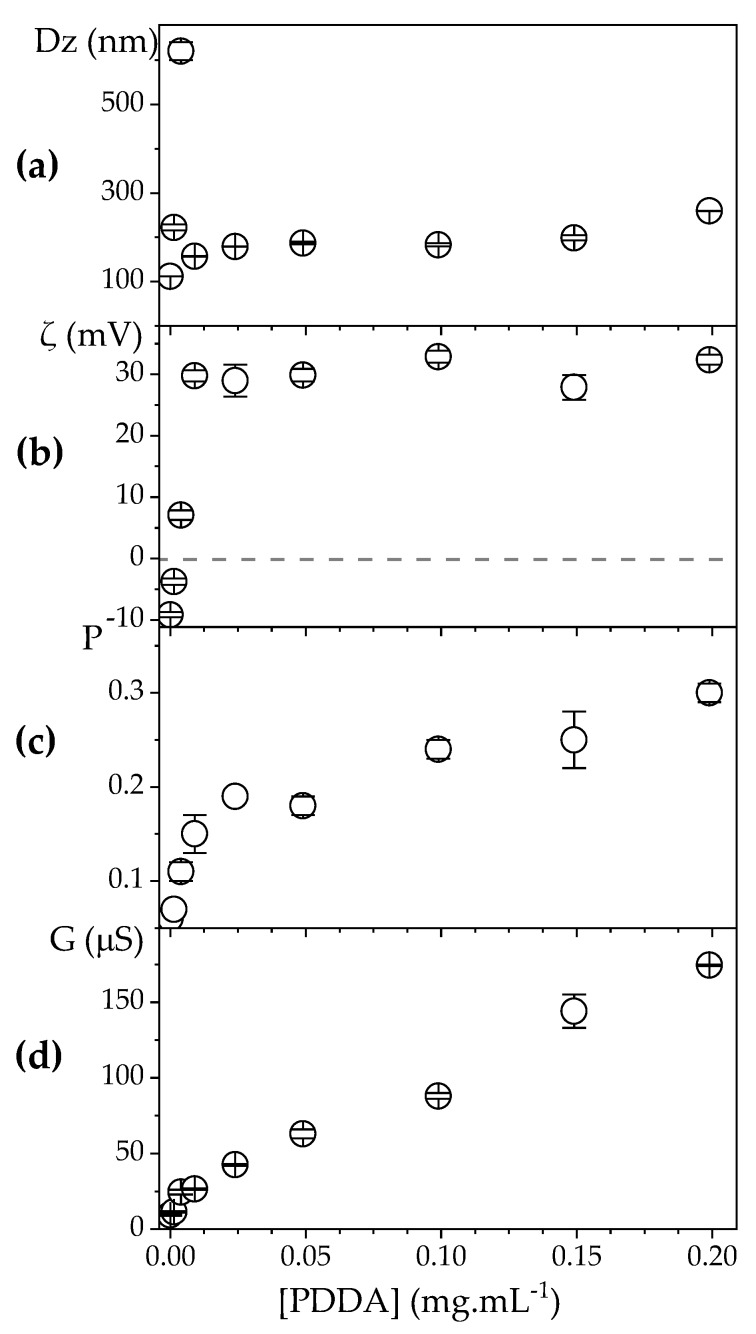
Effect of PDDA concentration on: (**a**) hydrodynamic diameter (Dz); (**b**) zeta potential (ζ); (**c**) polydispersity (P); (**d**) conductance (G) of PDDA/OVA NPs at [OVA] = 0.1 mg. mL^−1^. The dashed line indicates zeta-potential (ζ) = 0 occurring around [PDDA] = 0.004 mg·mL^−1^.

In order to evaluate the effect of [PDDA] on the physical properties of PDDA/OVA NPs at 0.1 mg·mL^−1^ OVA, the concomitant change in size (Dz), zeta-potential (ζ), polydispersity (P), and conductance (G) was obtained using the DLS apparatus. NPs size initially increased with PDDA concentration attaining a maximal value when ζ was zero meaning that absence of electrostatic repulsion between the NPs would cause loss of their colloidal stability (Figure 3a,b). Further increasing PDDA concentration to 0.01 mg·mL^−1^ re-stabilized the dispersion, yielding positive and relatively high zeta-potential (around 30 mV). This was the highest zeta-potential obtained over all ranges of PDDA concentrations tested. However, increasing [PDDA] above 0.01 mg·mL^−1^, an increase in the conductance (G) of the dispersions revealed that further incorporation of PDDA onto NPs did not occur (Figure 3d). Additional PDDA remained in the bulk solution increasing G. The poly-cation nature of PDDA has indeed been shown to result in increasing conductance with increases in PDDA concentration in water solutions [52]. Over a range of high [PDDA], above 0.2 mg·mL^−1^ PDDA, the polydispersity (P) of the dispersions increased (Figure 3c), possibly due to PDDA-induced bridging flocculation and PDDA/OVA NPs aggregation [39]. 

**Figure 4:**
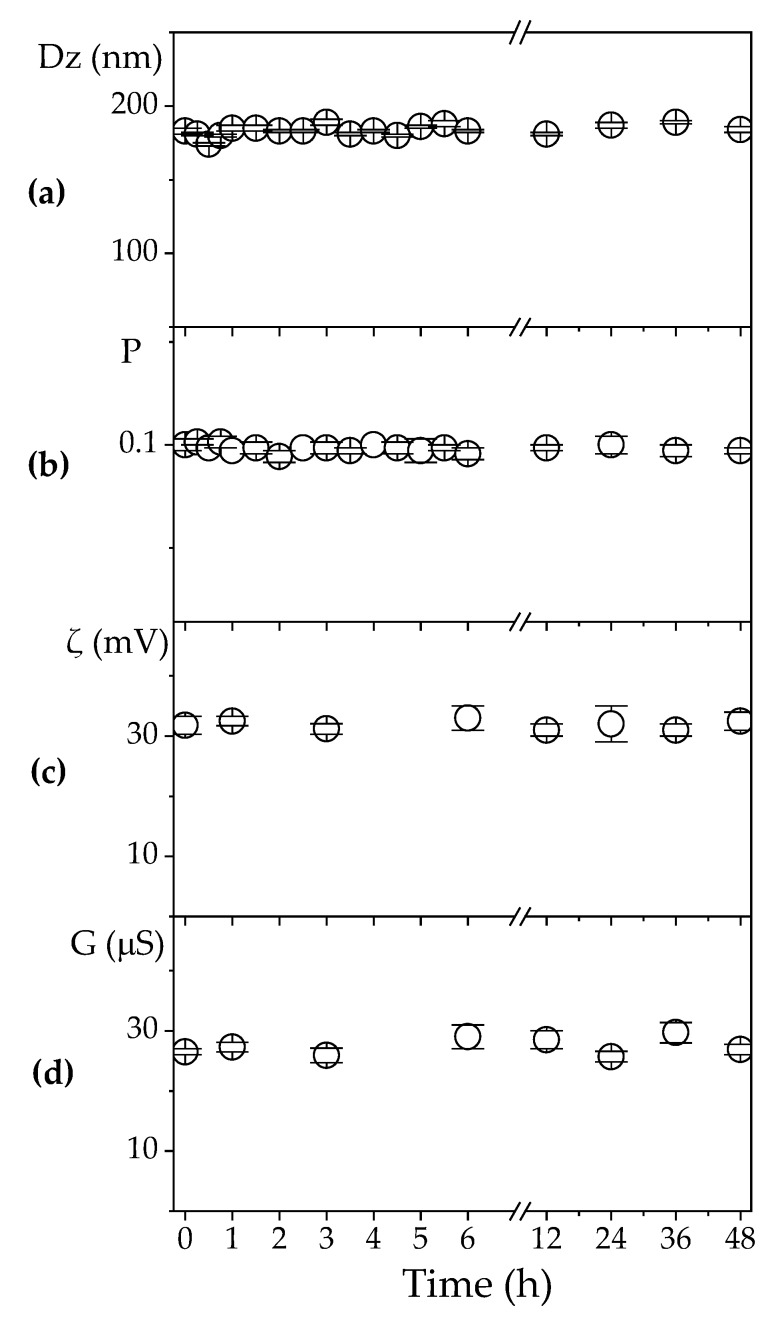
Effect of time on: (**a**) hydrodynamic diameter (Dz); (**b**) polydispersity (P); (**c**) zeta potential (ζ); (**d**) conductance (G) of PDDA/OVA NPs at [PDDA] = 0.01 mg·mL^−1^ and [OVA] = 0.1 mg. mL^−1^.

From the results in Figure 3, the best conditions for obtaining positively charged, stable, and nano-sized PDDA/OVA NPs became clear, namely, 0.1 and 0.01 mg·mL^−1^ for OVA and PDDA concentrations, respectively. At these concentrations, the usual cytotoxicity of cationic polymers is minimized [29]. The smallest concentration possible of the cationic polymer PDDA for obtaining the desired properties of the NPs was determined as 0.01 mg·mL^−1^ at 0.1 mg·mL^−1^ OVA (Figure 3). In Figure 4, using these same concentrations, the colloidal stability for the PDDA/OVA NPs in water was determined over the time remaining excellent during all the observation period (48 h) and possibly beyond (from visual and macroscopic observation). The NPs were stable colloids. Paul Dubin and co-workers also found good colloidal stability for PDDA/BSA NPs over 4 months [42].

### 3.2. Cytotoxicity of PDDA/OVA NPs Against Mammalian Cells in Culture

The cytotoxicity of the PDDA0.01/OVA0.1 formulation meaning 0.01 and 0.1 mg·mL-1 of PDDA and OVA concentrations was evaluated against two cell lines in culture, fibroblasts and macrophages, using two different techniques, the MTT assay and SEM.

**Figure 5:**
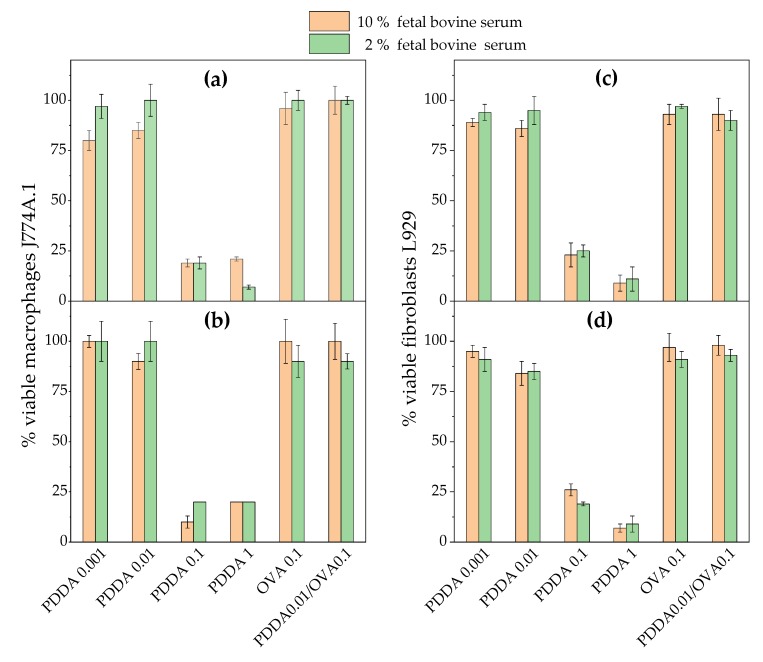
Cell viability (%) of macrophages (J774A.1) or fibroblasts (L929) in culture from the 3-(4,5-dimethylthiazol-2-yl)-2,5-diphenyl tetrazolium bromide (MTT) assay after incubation for 3 (**a**,**c**) or 24 h (**b**,**d**) with PDDA, OVA, and PDDA/OVA NPs. Concentrations are given in mg·mL^−1^. One should notice that the formulation used for the immunology experiments were 0.01 and 0.1 mg. mL^−1^ of PDDA and OVA, respectively.

In Figure 5, significant cytotoxicity for PDDA against the mammalian cells was obtained only above 0.01 mg·mL^−1^ for the two time points (3 and 24 h), and serum concentrations were tested (2% and 10% FBS). This agrees with data by Fischer and coworkers who determined low cytotoxicity for 0.01 mg·mL^−1^ PDDA against fibroblasts. Along similar lines, hemolytic effects of PDDA did not occur at such low concentration [53].

The use of two serum concentrations aimed at finding an eventual effect of serum proteins neutralization of the positive charges on PDDA contributing to diminished cytotoxicity. The results in Figure 5 showed that both serum concentrations had a similar effect on cytotoxicity against the two cell lines. Possibly, at 0.01 mg·mL^−1^ PDDA, BFS at 2% was enough to combine with all PDDA molecules so that PDDA alone was not available to combine with the cells. On the other hand, at higher [PDDA], PDDA molecules that did not combine with BFS would attach to the cells exerting their cytotoxic activity. The hemolytic activity of PDDA was reported as important only above [PDDA] = 1 mg·mL^−1^ [29,33,53].

In agreement with the present findings, Fischer and co-workers also reported low cytotoxicity of PDDA against fibroblasts at 0.01 mg·mL^−1^ using the MTT assay besides showing that PDDA was less toxic against the cells than PEI or PLL [29].

**Figure 6:**
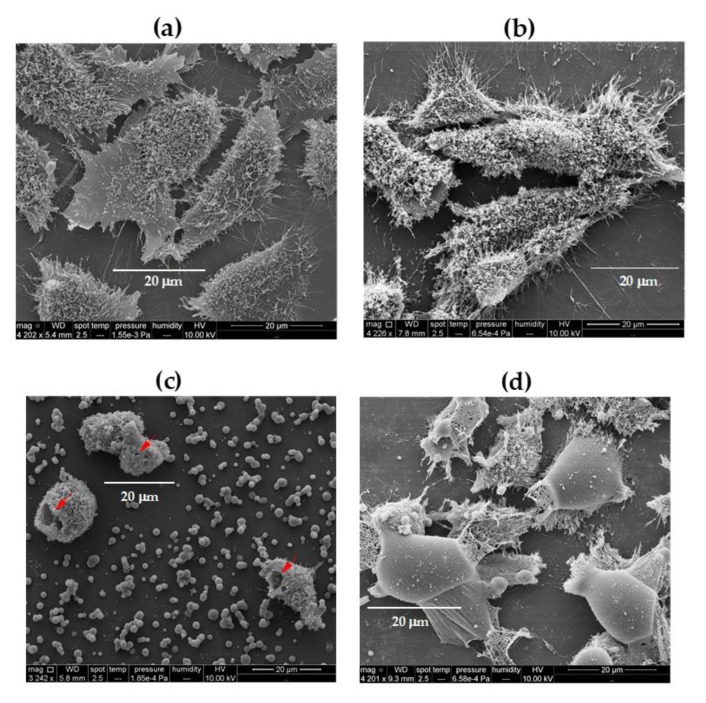
Scanning electron microscopy (SEM) of fibroblasts L929 incubated for 3 h with: (**a**) culture medium; (**b**) 0.01; (**c**) 0.1; (**d**) 1 mg·mL^−1^ PDDA. The red arrows indicate holes on the cells at 0.1 mg·mL^−1^ PDDA.

The effect of PDDA on morphology of the fibroblasts is presented in Figure 6. Holes could be seen on the cells indicating that the many debris observed in the SEM micrographs stemmed from the cell membrane, apparently withdraw from the cell by the cationic polymer PDDA. Similar cell debris were observed by Martinez et al. with 10 mM poly(allylamine hydrochloride) [31] and with linear or ramified PEI or PLL [30], suggesting a general feature of poly-cations acting by disruption of cell membranes. This also took place for multi-resistant bacteria submitted to PDDA assemblies with anionic carboxymethylcellulose (CMC) for which both the cell wall and the cell membrane were lysed allowing the leakage of intracellular compounds [32]. 

### 3.3. Immunoadjuvant Properties of PDDA/OVA NPs. 

**Figure 7:**
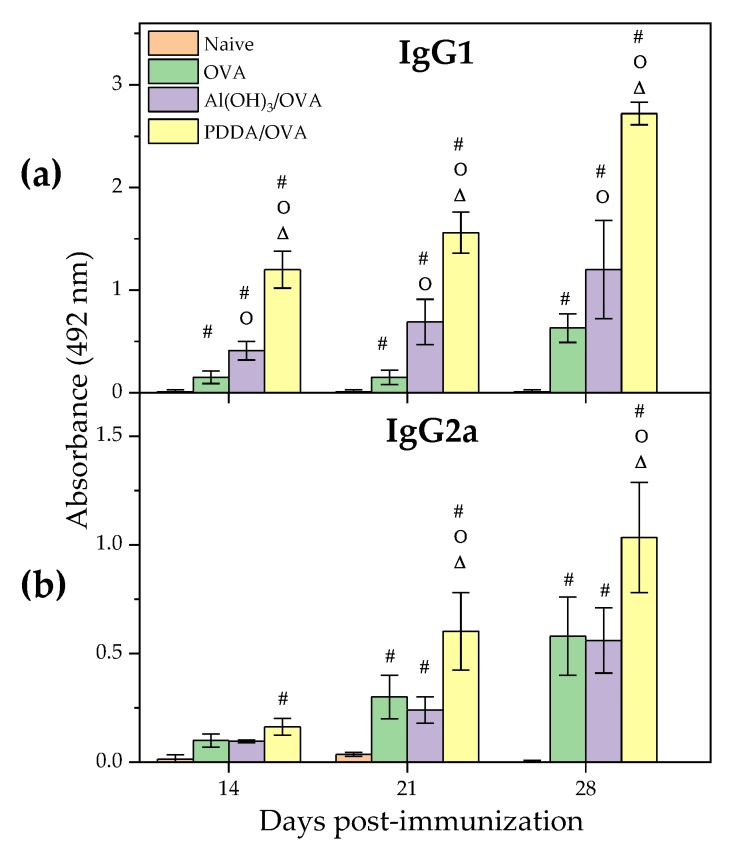
Humoral responses induced by OVA, Al (OH)_3_/OVA, or PDDA/OVA NPs at concentrations given in Table 1. Mean absorbance at 492 nm± standard deviation related to anti-OVA IgG1 and IgG2a antibodies production was determined over time after immunization of BALB/c mice from sera collected on days 14 and 21 post-immunization (primary response) or on day 28 (secondary response). The primary IgG1 production was determined at 1/256 serum dilution whereas the secondary one was obtained at 1/16348 dilution. The primary IgG2a production was determined at 1/8 serum dilution whereas the secondary one was obtained at 1/128 dilution. *p* < 0.05 compared to naive group (#), *p* < 0.05 compared to OVA group (o), *p* < 0.05 compared to Al(OH)_3_/OVA group (∆).

Adjuvants as Al(OH)_3_ usually improve the antigen-specific production of IgG1, whereas adjuvants as the cationic lipid dioctadecyldimethylammonium bromide (DODAB) dispersed as bilayer assemblies usually implement the antigen-specific production of IgG2a [8,15,20,25,27]. Figure 7 shows the humoral responses of mice challenged with Al(OH)_3_/OVA0.1 dispersion and PDDA0.01/OVA0.1 NPs as determined by ELISA for detection of IgG1 and IgG2a antibodies. The NPs displayed an immune response profile qualitatively very similar to the one of Al (OH)_3_ [54] yielding an expressive increase in IgG1 and a discrete increase in IgG2a production as usual for a Th-2 implemented response by an adjuvant. However, in quantitative comparison with Al(OH)_3_, PDDA as an adjuvant the NPs were more effective than Al(OH)_3_/OVA for implementing OVA-specific IgG1 production. In the literature, the size histogram for Al(OH)_3_ dispersion alone or carrying an antigen showed a broad size distribution with high polydispersity and low colloidal stability as depicted from the presence of precipitated material [17]. There was a much higher production of IgG1 than IgG2a, as depicted from the serum dilutions used to determine by ELISA both antibodies. Whereas the serum dilution used to determine the primary IgG1 production was 1/256, the one used to determine primary IgG2a production was 1/8 only. This means that the serum contained much larger IgG1 concentration than the one of IgG2a (Figure 7).

The advantages of the PDDA/OVA NPs are their nano-size (Figure 1 and Figure 2), high colloidal stability (Figure 4), and visual absence of precipitated material. Possible consequences in vivo would be the optimization of antigen presentation by APC at the lymph nodes. Indeed, Manolova and co-workers [55] found that nano-sized adjuvant/antigen assemblies directly delivered the antigen to the APC in the lymph nodes, whereas micro-sized ones stayed longer at the site of injection becoming mostly associated with dendritic cells (DC) from the site of injection. This could explain the higher production of IgG1 induced by PDDA/OVA NPs in comparison to Al(OH)_3_ /OVA. 

Other cationic polymers carrying antigen also improved the humoral response above the one obtained with the traditional Al(OH)_3._ Diverse forms of PEI elicited potent mucosal adjuvant activity for viral subunit glycoprotein antigens; a single intranasal administration of influenza hemagglutinin or herpes simplex virus type-2 (HSV-2) glycoprotein D with PEI elicited robust antibody-mediated protection also superior to the one by existing experimental mucosal adjuvants [36].

Using other injection modes, PEI administered subcutaneously with viral glycoprotein (HIV-1 gp140) also enhanced antigen-specific serum IgG production. PEI elicited higher titers of both antigen-binding and -neutralizing antibodies than alum in mice and rabbits, and induced an increased proportion of antibodies reactive with native antigen. In an intraperitoneal model, PEI recruited neutrophils followed by monocytes to the site of administration and enhanced antigen uptake by antigen-presenting cells [37].

Gold nanorods modified with cationic CTAB, PDDA, and PEI were used as promising DNA vaccine adjuvants for HIV-1 treatment and, curiously, also yielded high ratios IgG1/IgG2a. Among these three cationic molecules, PDDA was the one yielding the higher ratios [43]. Plebanski and coworkers designed magnetic lipoplexes for enhanced DNA vaccine delivery combining PEI, superparamagnetic iron oxide nanoparticles (SPIONs) with the mucoadhesive hyaluronic acid (HA) and a plasmid encoding a malaria antigen increasing IgG1, IgG2a, and IgG2b [10,18]. 

**Figure 8:**
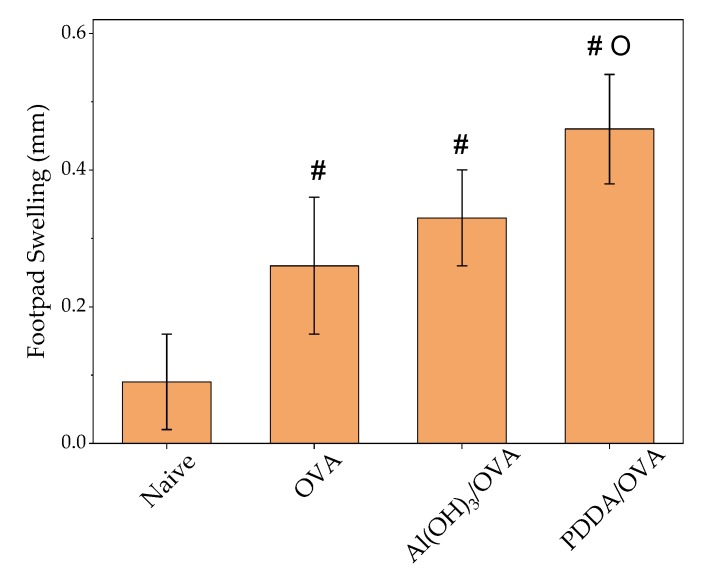
Mean footpad swelling ± standard deviation related to delayed-type hypersensitivity reaction of BALB/c mice immunized with OVA, Al(OH)_3_/OVA, or PDDA/OVA NPs at concentrations given on Table 1. *p* < 0.05 compared to naive group (#), *p* < 0.05 compared to OVA group (o).

In order to ascertain the cell-mediated immunity induced by the PDDA/OVA NPs, the delayed-type hypersensitivity reaction in immunized mice was determined. The DTH response induced by PDDA/OVA was low and equal to the one observed in mice for Al(OH)_3_/OVA (Figure 8). This reconfirms the Th-2 profile induced by PDDA/OVA NPs. Other Th-1 inducers such DODAB/OVA or DODAB/CpG/OVA gave footpad swellings above 1.25 mm in contrast to the value lower than 0.5 mm obtained for PDDA/OVA or Al(OH)_3_/OVA [8,9,12,15,17]. Thus, the low DTH response agreed with the predominant Th-2 profile for PDDA/OVA NPs. 

Recently, we described hybrid NPs made of poly (methyl methacrylate) (PMMA) in the presence of poly (diallyl dimethyl ammonium) chloride (PDDA) using dioctadecyl dimethyl ammonium bromide (DODAB) as an emulsifier and found that DODAB remains attached to the NPs core [56,57,58,59]. This system might bring interesting applications for antigen presentation since DODAB might eventually add the missing Th-1 response to the antigen-presenting NPs.

## 4. Conclusions

PDDA/OVA at 0.01 and 0.10 mg·mL^−1^ assembled as stable cationic nanoparticles in water with Dz = 170 nm ± 4, ζ = 30 mV ± 2, and P = 0.11 ± 0.01 effectively presented OVA to the immune system inducing a Th-2 type (humoral) response superior to the one induced by Al(OH)_3_. One of the most important advantages of the PDDA system as an adjuvant is its simplicity; it is a single-component adjuvant. In addition, at the low PDDA concentration used, cytotoxicity of the cationic polymer became neglectable. At and above 0.1 mg·mL^−1^, significant PDDA cytotoxicity against fibroblasts and macrophages in culture induced holes and cellular debris observable in SEM micrographs of the mammalian cells. PDDA/OVA NPs were nano-sized, stable, and well-dispersed in water without precipitated material. These properties would lead to in vivo optimization of antigen presentation by APC at the lymph nodes in contrast to the micro-sized Al(OH)_3_/OVA dispersion that would remain at the site of injection staying longer at the site of injection and stimulating local dendritic cells. Determination of delayed-type hypersensitivity (DTH) reaction from footpad swelling revealed the low cellular response induced by PDDA/OVA similarly to Al(OH)_3_ /OVA. In the future, it will be interesting to evaluate combinations of the antigen with NPs carrying both PDDA and elicitors of the Th-1 response such as cationic lipid and/or CpG. For continuing this work, the mechanism of PDDA-induced cell death might be investigated from the morphology of the cell nucleus using a fluorescent label to discriminate between necrosis or apoptosis. The location of nanoparticles inside the cell will have to be assessed using an ovalbumin fluorescent label and the CD8+/CD4+ cell proliferation from flow cytometry and cytokines profile determination will reconfirm the Th-1/Th-2 response induced by PDDA/OVA NPs.

## Figures and Tables

**Table 1:** Composition of solutions or dispersions in water used for immunization of four groups of five mice.

Group	Solution or Dispersion	[OVA] mg·mL^−1^	[Al(OH)_3_] mg·mL^−1^	[PDDA] mg·mL^−1^
1	Milli-Q water	0	0	0
2	OVA	0.1	0	0
3	Al(OH)_3_/OVA	0.1	0.1	0
4	PDDA/OVA	0.1	0	0.01
